# Immobilized Fe_3_O_4_-Polydopamine-*Thermomyces lanuginosus* Lipase-Catalyzed Acylation of Flavonoid Glycosides and Their Analogs: An Improved Insight Into Enzymic Substrate Recognition

**DOI:** 10.3389/fbioe.2021.798594

**Published:** 2021-11-16

**Authors:** Zhaoyu Wang, Yang Li, Mingyi Li, Xiaohui Zhang, Qingxia Ji, Xiaojuan Zhao, Yanhong Bi, Si Luo

**Affiliations:** School of Life Science and Food Engineering, Huaiyin Institute of Technology, Huai’an, China

**Keywords:** flavonoid glycosides, immobilized enzyme, magnetic nanoparticle, *Thermomyces lanuginosus* lipase, substrate recognition

## Abstract

The conversion of flavonoid glycosides and their analogs to their lipophilic ester derivatives was developed by nanobiocatalysts from immobilizing *Thermomyces lanuginosus* lipase (TLL) on polydopamine-functionalized magnetic Fe_3_O_4_ nanoparticles (Fe_3_O_4_-PDA-TLL). The behavior investigation revealed that Fe_3_O_4_-PDA-TLL exhibits a preference for long chain length fatty acids (i.e., C10 to C14) with higher reaction rates of 12.6–13.9 mM/h. Regarding the substrate specificity, Fe_3_O_4_-PDA-TLL showed good substrate spectrum and favorably functionalized the primary OH groups, suggesting that the steric hindrances impeded the secondary or phenolic hydroxyl groups of substrates into the bonding site of the active region of TLL to afford the product.

## Introduction

Ever since the groundbreaking work of Klibanov in the early 1980s (Zaks and Klibanov, 1984), nonaqueous enzymology has represented a significant extension of organic synthetic processes that come with more challenges—or that is even impossible—compared with traditional chemical approaches (Hughes and Lewis, 2018; van Schie et al., 2021). Studies over the past 30 years have revealed that enzymatic catalysis shows unusual abilities and can be a powerful tool for synthesizing fine chemicals in the pharmaceutical, food, and cosmetic industries (Sheldon and Pereira, 2017; SÁ et al., 2017).

Flavonoid glycosides and their analogs are a large group of well-known natural plant secondary metabolites that are of great academic and industrial interest because of their potentially beneficial biological, pharmacological, and medicinal properties (Chen et al., 2018; Yang et al., 2018). Because of their active polyhydroxyl structure, however, these compounds usually exert a moderately hydrophilic nature and instability, which limits their primary application in lipophilic systems (Xin et al., 2018; Yang et al., 2018). Recently, selective structural and functional modifications of natural flavonoid glycosides and their analogs based on their basic flavonoid skeletons by fatty acid acylation have been demonstrated not only to improve their physicochemical properties, but also to enrich new drug design and discovery strategies (Newman and Cragg, 2016). For example, de Araújo et al. recently comprehensively reviewed the preferable biological benefits of the flavonoid ester derivates (de Araújo et al., 2017).

Over the past few years and compared with the arduous and harsh chemical method, an enzymatic methodology in nonaqueous systems to form diverse natural product acylates has proved to be greatly beneficial, featuring mild condition processing, environmental benignancy, and adjustable selectivity (Sheldon and Pereira, 2017). However, the drawbacks of the enzyme catalyst, such as poor organic solvent tolerance, low reusability, and unsatisfied continuous operability in industrial enzymatic processes, may still be unavoidable (Gonçalves Filho et al., 2019; Liang et al., 2020). In this context, chemists have been committed to overcoming these shortcomings by exploring solvent engineering, substrate engineering, or enzyme engineering strategies. Recently, with the rapid development of nanotechnology, magnetic nanoparticles incorporated with biodegradable mussel-inspired polydopamine (PDA) nanocarriers, cellulose nanocrystals (CNCs), and biopolymer nanogels have generated intense interest and been successfully introduced to immobilize enzymes; these processes are characterized by superior enzyme loading capacity, high enzyme activity recovery, long-term operational stability, and facilitated separation for reuse (Bilal et al., 2020; Wang et al., 2020b; Liang et al., 2020). For example, a considerable amount of studies on constructing enzyme-immobilized magnetic nanosystem *via* a simple method have been successfully performed by Lou et al., and the results showed that both the magnetic Fe_3_O_4_-CNC-papain and Fe_3_O_4_-PDA-lipase improved the performance of enhanced enzyme loading, catalytic efficiency, and solvent tolerance compared with their free forms (Cao et al., 2016; Liang et al., 2020).

Up to now, although magnetic nanostructures have drawn extensive attention and much work has been reported, no substantial empirical rules can be followed to facilitate the immobilization of various enzymes and guide their application in the manufacturing of enzyme-processed products (Bi et al., 2020; Liang et al., 2020). Most of the studies were usually carried out using a trial-and-error method. To some extent, this phenomenon might be attributed to the absence of a detailed discussion of the molecular mechanisms of catalytic behaviors of the nanocatalyst. For these reasons, in the current study, the acylation of flavonoid glycosides and their analogs catalyzed with Fe_3_O_4_-PDA-*Thermomyces lanuginosus* lipase (Fe_3_O_4_-PDA-TLL) was selected as a model reaction to elucidate the catalytic performance of the nanocatalyst, here based on revealing the molecular basis of substrate recognition specificity by using a substrate engineering strategy; doing this may provide some valuable information for constructing an enzyme-immobilized magnetic nanosystem.

## Materials and Methods

### Catalysts and Chemicals

CALB (powder, lipase from *Candida antarctica* B), TLL (powder, lipase from *Thermomyces lanuginosus*), RML (powder, lipase from *Rhizomucor miehei*), TLIM (lipase from *Thermomyces lanuginosus*, immobilized on granulated silica), and MML (powder, lipase from *Mucor miehei*) were purchased from Novozymes Co., Ltd, China. PEL (powder, lipase from *Penicillium expansum*) and ANL (powder, lipase from *Aspergillus niger*) were obtained from Leveking Bioengineering Co., Ltd, Shenzhen, China. Polydatin, arbutin, helicid, dihydromyricetin, dopamine hydrochloride, and 2-methyltetrahydrofuran (MeTHF) were provided by Aladdin. Hyperoside, vinyl sorbate, and vinyl laurate were obtained from Sigma-Aldrich. Vinyl butyrate, vinyl hexanoate, vinyl octanoate, vinyl decanoate, vinyl myristate, vinyl palmitate, vinyl pivalate, vinyl 2-ethylhexanoate, vinyl stearate, vinyl benzoate, vinyl crotonate, and vinyl undecenoate were from TCI. Quercimetrin, isoquercetin, astragaline, myricetrin, taxifolin, gastrodin, and baicalin were from Yihe Biotechnology Co., Ltd, Shanghai, China. All other chemicals were also from commercial sources and of the highest purity available.

### Preparation of Immobilized Lipase

Magnetic Fe_3_O_4_ nanoparticles were fabricated by a coprecipitation method per the methods reported previously (Abbaszadeh and Hejazi, 2019). To prepare PDA-coated Fe_3_O_4_, a certain amount of dopamine hydrochloride was added into deionized water containing an equivalent molar quantity of magnetic Fe_3_O_4_ nanoparticles, which were dispersed and sonicated for 10 min before usage. The pH of the mixture was adjusted to 8.5 by the addition of a 1.5 M NaOH solution. After stirring for 24 h, a polydopamine-coated magnetic nanoparticle (Fe_3_O_4_-PDA) was formed and collected through a permanent magnet and washed three times with deionized water. In the next step, an aqueous solution of TLL was prepared by dissolving the TLL powder in phosphate buffer (260 mg/ml). Then, 2.4 ml of a TLL solution and 0.4 g of Fe_3_O_4_-PDA were added into 12 ml of a phosphate buffer (50 mM, pH 8.5) in a 25°C water bath. After stirring at 200 rpm for 4.0 h, the immobilized TLL was separated and continuously washed with the above buffer until no TLL was detected in the supernatant. The TLL-loaded Fe_3_O_4_-PDA was named Fe_3_O_4_-PDA-TLL.

### Assaying of Enzyme Esterification Activity

An immobilized TLL lipase assay was performed using the *p*-nitrophenyl palmitate (*p*-NPP) method (Soni et al., 2018). The substrate solution contained 30 mg of *p*-NPP in 10 ml of isopropanol. To initiate the reaction, 0.1 g of Fe_3_O_4_-PDA-TLL was added to the mixture of the substrate solution (0.1 ml) and Tris-HCl buffer (0.9 ml, 50 mM, pH 8.0) and incubated at 37°C for 10 min; here, absorbance was measured at 410 nm in a spectrophotometer. One unit of activity (U) was defined as the amount of enzyme required to produce 1.0 μmol *p*-nitrophenol (*p*-NP) in 1.0 min under the above conditions. The specific activities of Fe_3_O_4_-PDA-TLL, TLL, TLIM, CALB, RML, MML, ANL, and PEL were 8,022, 3,921, 9,750, 10,200, 2,575, 2033, 2,100, and 1990 U/g, respectively.

### Enzymatic Synthesis of Ester Derivatives

The reaction was carried out in 3.0 ml pure organic solvent, which was dried over 4 Å molecular sieves before usage, with each containing 0.03 mmol substrate, 0.33 mmol vinyl decanoate, and 180 mg Fe_3_O_4_-PDA-TLL. The reaction mixture was put in a 10 ml Erlenmeyer shaking flask at 55°C and at 200 rpm. Aliquots were withdrawn from the reaction mixture at specified time intervals and then diluted 50-fold with mobile phase before being analyzed by HPLC. The above experiment was conducted in triplicate.

### HPLC Analysis of Ester Derivatives

The reaction mixtures were analyzed by a HPLC (Agilent 1,200) system containing an XDB-C18 column (4.6 mm × 250 mm, 5 μm, Agilent) and an ultraviolet detector. Samples (20 μL) were eluted by a mixture of water and methanol at a rate of 1.0 ml/min, and detected at wavelengths of 360 (quercimetrin, isoquercetin, hyperoside, astragaline, and myricetrin), 282 (arbutin), 220 (gastrodin), 278 (baicalin), 270 (helicid), and 280 nm (polydatin, dihydromyricetin, and taxifolin), respectively (see Supplementary material).

### Determination of the Chemical Structure of Ester Derivatives

After the reactions, the immobilized enzyme was removed by filtration. Then, the filtrates were evaporated under vacuum. The products were separated and purified by silica gel chromatography with an eluent consisting of petroleum ether (PE) and ethyl acetate (EA) for the mobile phase. The chemical structures of the purified products were determined by ^1^H NMR and ^13^C NMR in DMSO-*d*_6_ using a Brucker DRX-400 NMR spectrometer at 400 and 100 MHz, respectively. The results from the NMR spectroscopy are given in the supplementary material.

## Results and DISCUSSION

### Influence of Enzyme Source

The catalytic behavior of the enzyme depends strongly on its source, which is well-known as the vital influence factor of the catalytic procedure and manipulates the catalytic activity, selectivity, and effectiveness (Littlechild, 2017; Gonçalves Filho et al., 2019; Wang et al., 2020a). Among the enzymes widely used in acylating polyhydroxy compounds, lipases have been shown to have preferential catalytic characteristics over the other kinds of enzymes (Xin et al., 2019). Therefore, comparative experiments were initially devoted to an enzyme screening based on the decanoylation of polydatin as a model reaction. The results are summarized in Table1.

**TABLE 1:** Regioselective decanoylation of polydatin catalyzed by various lipases.

Enzyme	Source	V_0_ (mM/h)	Time (h)	C*^a^* (%)	OH*^b^*-regioselectivity*^c^* (%)
TLL	*Thermomyces lanuginosus*	3.7 ± 0.1	9.0	35.4 ± 0.3	100
Fe_3_O_4_-PDA-TLL	*Thermomyces lanuginosus*	12.6 ± 0.3	14.0	98.7 ± 0.4	100
TLIM	*Thermomyces lanuginosus*	9.7 ± 0.2	14.0	94.1 ± 0.3	100
RML	*Rhizomucor miehei*	2.1 ± 0.1	6.0	20.5 ± 0.4	100
CALB	*Candida antarctica* B	3.8 ± 0.2	14.0	31.6 ± 0.5	100
PEL	*Penicillium expansum*	2.1 ± 0.2	6.0	17.3 ± 0.3	100
ANL	*Aspergillus niger*	2.4 ± 0.1	6.0	21.8 ± 0.4	100
MML	*Mucor miehei*	0.4 ± 0.1	6.0	11.1 ± 0.5	100
Control	―	n.d*^d^*	4.0	―	―

The reactions were carried out at 55°C and 200 rpm by adding 0.03 mmol polydatin, 0.33 mmol vinyl decanoate, 180 mg enzyme into 3.0 ml anhydrous MeTHF.

a Maximum substrate conversion.

b Primary hydroxyl.

c Regioselectivity was defined as the ratio of the concentration of the indicated product to that of all the products formed.

d Not detected.

For the enzymes, we screened six lipases, either in their free form or supported on different carriers; as expected, two supported lipases possessed an excellent ability to acylate polydatin with vinyl decanoate. For example, the nanocatalyst TLL, which was immobilized on nano-Fe_3_O_4_-PDA (Fe_3_O_4_-PDA-TLL), afforded the highest initial reaction rates of 12.6 mM/h and substrate conversions of 98.7%, while the other examined free enzymes were hardly available. Moreover, TLL immobilized on magnetic Fe_3_O_4_-PDA makes the isolation of product very easy as the catalyst can be removed from the reaction with the help of a magnet.

Regarding the position of the acylation, all of the investigated lipases preferentially acylated the more active and less steric hindrance primary hydroxyl group in polydatin and formed monoester derivatives by NMR spectra analysis. Particularly for *T. lanuginosus* lipase, the immobilized supports, including Fe_3_O_4_-PDA and granulated silica, did not change the enzyme’s selectivity toward the OH position. In the acylation of polyhydroxy nucleosides reported by Li and our group (Li et al., 2009a; Wang et al., 2011), however, TLIM was been found to be superior to other enzymes when it came to functionalizing its favorable secondary hydroxyl group. X-ray crystallographic studies have shown that the amphiphilic active center of TLL contains a large hydrophobic acyl chain-binding groove and a smaller hydrophilic pocket in which the alcohol moiety and Tyr 21 bind (Noinville et al., 2002). Therefore, this distinctive binding model might be beneficial for increasing the hydrogen-bond interactions between the phenolic hydroxyl groups of the substrate with amino acid residue Tyr 21, thus stabilizing the conformation of the acylation transition state and enhancing the primary hydroxyl regioselectivity.

### Influence of Reaction Medium

The reaction solvents used in nonaqueous biocatalysis can modulate the catalyst’s catalytic activity, selectivity, substrate specificity, and stability (Pätzold et al., 2019; van Schie et al., 2021). However, no theory of experience can be applied for solvent choice, even to this day, and trial-and-error experiments still are inevitable for the given biocatalytic reaction. As can be seen in Table 2, apart from the case of polar DMSO (which can lead to an increase in the internal rigidity of the enzyme protein and result in enzyme inactivation by stripping the essential water layer off the enzyme molecules), Fe_3_O_4_-PDA-TLL presented substantial acylation activity and an excellent conversion (90.0–98.7%) in all the tested media. Compared with the other traditional organic solvents, the biomass-derived MeTHF always displayed excellent biocompatibility and contributed the highest initial rate and polydatin conversion.

**TABLE 2:** Effect of organic solvents on Fe_3_O_4_-PDA-TLL-catalyzed decanoylation of polydatin.

Solvent	Log *p*	V_0_ (mM/h)	Time (h)	C (%)	OH*^a^*-regioselectivity (%)
Dioxane	−1.10	9.4 ± 0.3	14.0	94.7 ± 0.05	100
DMSO	−1.00	―	15.0	3.1 ± 0.1	100
Acetone	−0.23	11.3 ± 0.5	14.0	98.0 ± 0.4	100
THF	0.49	10.8 ± 0.4	14.0	98.2 ± 0.3	100
*t*-Butanol	0.60	7.2 ± 0.2	16.0	90.0 ± 0.5	100
MeTHF	0.99	12.6 ± 0.6	14.0	98.7 ± 0.3	100

The reactions were carried out at 55°C and 200 rpm by adding 0.03 mmol polydatin, 0.33 mmol vinyl decanoate, 180 mg Fe_3_O_4_-PDA-TLL, into 3.0 ml anhydrous solvent.

a Primary hydroxyl.

However, the influence of thermodynamic parameter log *p* on enzyme behavior is rather unpredictable, in one case possessing a low log *p* value of −1.1 (e.g., dioxane); here, the catalytic efficiency may be satisfied, yet in another one (e.g., DMSO), the opposite may be observed. This is similar to the enzyme-mediated acylation nucleosides found by our group (Bi et al., 2014). In addition to the solvent polarity, a computer simulation study revealed that different solvent molecule with various spatial structures and sizes would exert a different ability to occupy the substrate biding site in active region of the enzyme (Yang et al., 2004), thus leading to further disruption of the enzymatic process.

### Influence of the Acyl Donor

A variety of acyl donors, such as saturated, unsaturated, branched, or aromatic, were chosen to evaluate the recognition regularity of the immobilized Fe_3_O_4_-PDA-TLL. Among the acyl donors investigated, 11 of the 14 vinyl esters could be introduced into polydatin, and the regioselectivity was overwhelming toward the primary hydroxyl position. Interestingly, when straight-chain saturated fatty acid vinyl esters (C4–C18) (Entries 1, 4, 7, 9, and 11–14, Table 3) were used for polydatin ester synthesis, Fe_3_O_4_-PDA-TLL may exhibit a slight preference to the long-chain fatty acids (e.g., C10 to C14) with the relative higher reaction rates of 12.6–13.9 mM/h. The reason might be attributed to the hydrophobic, crevice-like binding site of the *T. lanuginosus* lipase (Pleiss et al., 1998), in which in the longer the carbon chain of the acyl donor, a stronger hydrophobic interaction was produced, thus being more favorable for forming an acylation transition state and improving the enzymatic catalytic efficiency.

**TABLE 3:** Effect of acyl donor structure on the acylation of polydatin catalyzed by Fe_3_O_4_-PDA-TLL.

Entry	Acyl donor	Acyl group	V_0_ (mM/h)	Time (h)	C (%)	OH*^a^*-regioselectivity (%)
1	Vinyl butyrate	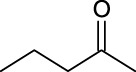	9.8 ± 0.4	14.0	98.1 ± 0.1	100
2	Vinyl crotonate	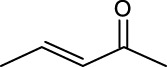	4.7 ± 0.1	18.0	90.1 ± 0.2	100
3	Vinyl pivalate	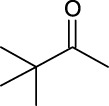	n.d	6.0	―	―
4	Vinyl hexanoate	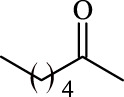	12.3 ± 0.4	14.0	98.1 ± 0.4	100
5	Vinyl sorbate	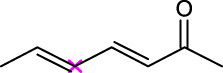	6.3 ± 0.2	18.0	91.3 ± 0.3	100
6	Vinyl benzoate	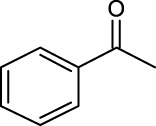	n.d	6.0	―	―
7	Vinyl octanoate	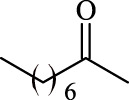	12.4 ± 0.3	14.0	98.4 ± 0.2	100
8	Vinyl 2-ethylhexanoate	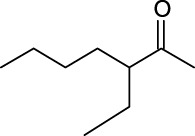	n.d	6.0	―	―
9	Vinyl decanoate	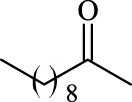	12.6 ± 0.4	14.0	98.7 ± 0.5	100
10	Vinyl undecenoate	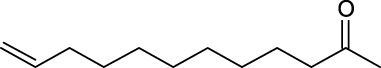	13.9 ± 0.5	14.0	98.8 ± 0.6	100
11	Vinyl laurate	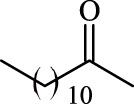	13.7 ± 0.6	14.0	99.1 ± 0.5	100
12	Vinyl myristate	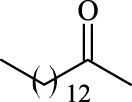	12.8 ± 0.3	14.0	97.5 ± 0.3	100
13	Vinyl palmitate	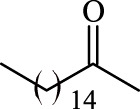	12.6 ± 0.2	14.0	97.5 ± 0.4	100
14	Vinyl stearate	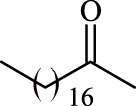	11.2 ± 0.3	14.0	96.0 ± 0.5	100

The reactions were carried out at 55°C and 200 rpm by adding 0.03 mmol polydatin, 0.33 mmol vinyl donor, 180 mg Fe_3_O_4_-PDA-TLL, into 3.0 ml anhydrous MeTHF.

a Primary hydroxyl.

As predicted, no reactions occurred using branched vinyl donors such as vinyl pivalate (Entry 3, Table 3) and vinyl 2-ethylhexanoate (Entry 8, Table 3) because of the presence of a severe steric hindrance between the branched chain of the acyl donor and the large hydrophobic acyl chain-binding pocket (Yang et al., 2010). For the unsaturated and aromatic acyl donors, a significant variation of the initial rates and conversions were obtained during acylation. As shown in Table 3, Fe_3_O_4_-PDA-TLL displayed no or low activity using vinyl benzoate, vinyl crotonate, and vinyl sorbate (Entries two and 5–6, Table 3), while the reactions were smooth in the reaction systems containing vinyl undecenoate (Entry 10, Table 3). The resonance effect stemming from the interaction between the conjugated *α*-double bond of the acyl chain and carbonyl carbon may strengthen the electron cloud density of the carbonyl group and reduce the flexibility of the aliphatic chain (Kobayashi et al., 2003), thereby resulting in more difficulty in the conformation of an acylation transition state. When the C-C double bond in the acyl moiety is far away from the carbonyl group, this resonance effect on acylation is insignificant; in addition, the performance of the Fe_3_O_4_-PDA-TLL was similar to those in lauroylation (Entry 13, Table 3).

### Influence of the Substrate Structure

For an in-depth insight into the universal applicability of the immobilized nanocatalyst, 12 flavonoid glycosides and their analogs possessing various positions and numbers of primary, secondary, and tertiary hydroxyl requirements for regioselective catalysis by Fe_3_O_4_-PDA-TLL were established. As can be seen from Table 4, eight flavonoid glycosides (Entries 1–4 and 8–11, Table 4) could be converted into the desired products in excellent conversion (94.1–99.7%), indicating the good substrate spectrum of the immobilized Fe_3_O_4_-PDA-TLL.

**TABLE 4:** Effect of chemical construction of substrate on the acylation catalyzed by Fe_3_O_4_-PDA-TLL.

Entry	Substrate	Structure	V_0_ (mM/h)	Time (h)	C (%)	OH*^a^*-regioselectivity (%)
1	Quercimetrin	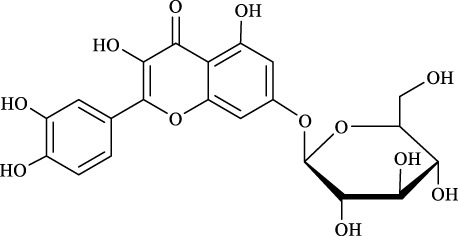	6.5 ± 0.2	16.0	94.1 ± 0.5	100
2	Isoquercetin	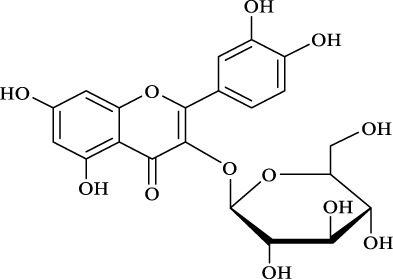	6.8 ± 0.3	16.0	95.1 ± 0.3	100
3	Hyperoside	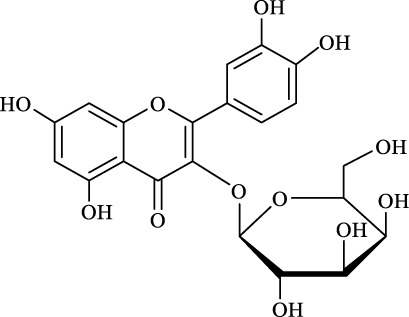	6.5 ± 0.3	16.0	97.5 ± 0.7	100
4	Astragaline	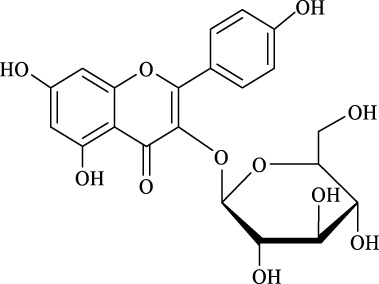	6.4 ± 0.3	16.0	95.3 ± 0.5	100
5	Myricetrin	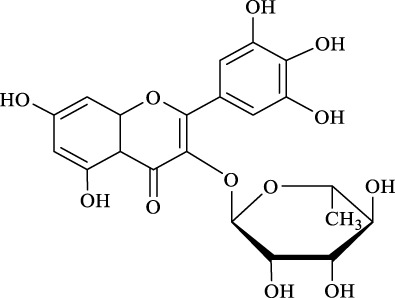	n.d	8.0	―	―
6	Dihydromyricetin	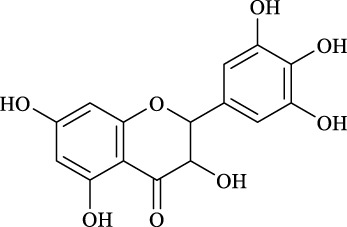	n.d	8.0	―	―
7	Taxifolin	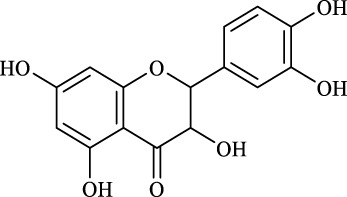	n.d	8.0	―	―
8	Gastrodin	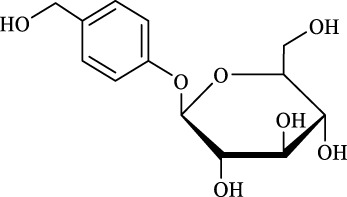	13.0 ± 0.5	10.0	99.7 ± 0.6	82
9	Polydatin	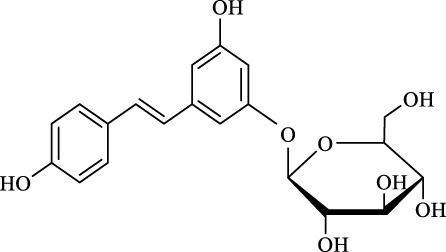	12.6 ± 0.4	12.0	98.7 ± 0.5	100
10	Helicid	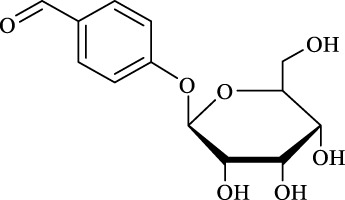	14.6 ± 0.5	10.0	99.3 ± 0.5	100
11	Arbutin	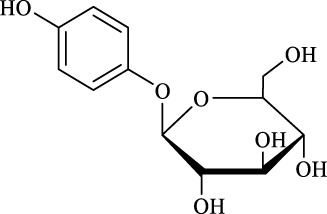	14.5 ± 0.3	14.0	99.6 ± 0.4	100
12	Baicalin	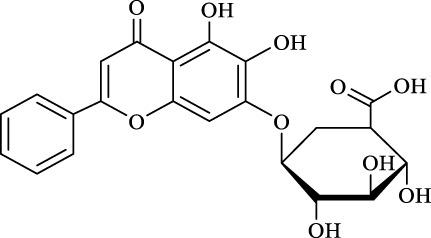	n.d	8.0	―	―

The reactions were carried out at 55°C and 200 rpm by adding 0.03 mmol polydatin, 0.33 mmol vinyl decanoate, 180 mg Fe_3_O_4_-PDA-TLL, into 3.0 ml anhydrous MeTHF.

a Primary hydroxyl.

Regarding the regioselectivity, all the finished acylations took place in the primary OH of the glucose moiety of the examined acylating substrates and produced monoester derivatives, except for the case of gastrodin, which has two more active primary OH and afforded the domain diester product with a regioselectivity of 82% (Entry 8, Table 4). However, the reaction rates were significantly higher for gastrodin, polydatin, helicid, and arbutin (Entries 8–11, Table 4) than for the other active compounds (Entries 1–4, Table 4). The reason might be that their relatively simple aglycone structures may enter into the active site more easily to attack the acyl-enzyme intermediate and prepare various glycoside ester derivatives, thus shortening the reaction time.

Surprisingly, although baicalin (Entry 12, Table 4) seemed to be less hindered than quercimetrin, isoquercetin, and hyperoside (Entries 1–3, Table 4) because of the presence of more phenolic hydroxyl groups in the aglycones in latters, no esterification product was found in the acylation. The differences in the spatial structure among them derived from the different bonding paths between the aglycone and glucose moiety may be responsible for this phenomenon (Li et al., 2009b). To verify the possibility of secondary OH groups acylated in the tested glycosides, their aglycone analogs, such as dihydromyricetin (Entry 6, Table 4) and taxifolin (Entry 7, Table 4), were selected as the acylation substrates. However, Fe_3_O_4_-PDA-TLL did not display the capacity for acylating the secondary OH groups in not only the glucose moiety, but also the aglycone structure. Most studies have suggested that the steric hindrance in the substrate structure blocked the secondary OH group in the bonding site to form the tetrahedral intermediate and product (Li et al., 2009b; Bi et al., 2014).

## Conclusion

In the present work, more insight into the substrate recognition law of the nanobiocatalyst of Fe_3_O_4_-PDA-TLL through a rational substrate engineering strategy was gained. The unexpected yet satisfactory results improved and extended the application of the magnetic nanoparticles for immobilizing TLL lipase in regioselectively acylating active polyhydroxy natural compounds in nonaqueous systems, which was demonstrated by receiving the higher catalytic activity, excellent selectivity, and good substrate specificity of Fe_3_O_4_-PDA-TLL. Based on these observations, the biocompatible Fe_3_O_4_ magnetic nanoparticles modified by biodegradable polydopamine (Fe_3_O_4_-PDA) can act as an alternative immobilized support for useful enzymes, indicating great potential for industrial applications.

## Data Availability

The original contributions presented in the study are included in the article/Supplementary Material, further inquiries can be directed to the corresponding author.
