# Supplementary material for: Immobilized Fe3O4-Polydopamine-Thermomyces lanuginosus Lipase-Catalyzed Acylation of Flavonoid Glycosides and Their Analogs: An Improved Insight Into Enzymic Substrate Recognition
Source: Front Bioeng Biotechnol. 2021 Nov 16;9:798594. doi: 10.3389/fbioe.2021.798594 (PMC8636704; doi:10.3389/fbioe.2021.798594)
Supplement: Supplementary file 1 [file DataSheet1.PDF]

Supplementary material

Immobilized Fe_3O_4-Polydopamine-*Thermomyces lanuginosus* Lipase-catalyzed Acylation of Flavonoid Glycosides and their Analogs: An Improved Insight into Enzymic substrate Recognition

Zhaoyu Wang, Yang Li, Mingyi Li, Xiaohui Zhang, Qingxia Ji, Xiaojuan Zhao, Yanhong Bi, Si Luo*

School of Life Science and Food Engineering, Huaiyin Institute of Technology, Huai'an 223003, China

* Correspondence: xy_7881@126.com (Y. Bi)

HPLC Analysis

The volumetric ratio of methanol to water and the retention time for polydatin and its *O*-monoester were 60/40, 2.54, and 4.01 min (butyrylation), 60/40, 2.63, and 3.87 min (crotonylation), 60/40, 2.55, and 7.34 min (hexanoylation), 60/40, 2.59, and 5.27 min (sorboylation), 80/20, 2.28, and 3.34 min (octanoyltion), 80/20, 2.31, and 4.93 min (decanoylation), 80/20, 2.29, and 4.65 min (undecenoylation), 90/10, 2.31, and 8.19 min (lauroylation), 90/10, 2.24, and 4.41 min (myristoylation), 90/10, 2.26, and 6.57 min (palmitoylation), 90/10, 2.23, and 9.03 min (stearoylation), respectively.

The volumetric ratio of methanol to water and the retention time for substrate and its *O*-*O*-decanoyl monoester were 80/20, 2.51, and 7.16 min (quercimetrin), 80/20, 2.52, and 7.15 min (isoquercetin), 80/20, 2.50, and 6.11 min (hyperoside), 80/20, 2.50, and 4.48 min (astragaline), 80/20, 2.52, and 7.70 min (helicid), 80/20, 2.59, and 6.01 min (arbutin), respectively. The volumetric ratio of methanol to water and the retention time for gastrodin and its *O*-decanoyl diester were 80/20, 2.51, and 6.67 min, respectively.

Structure determination of the esters by NMR

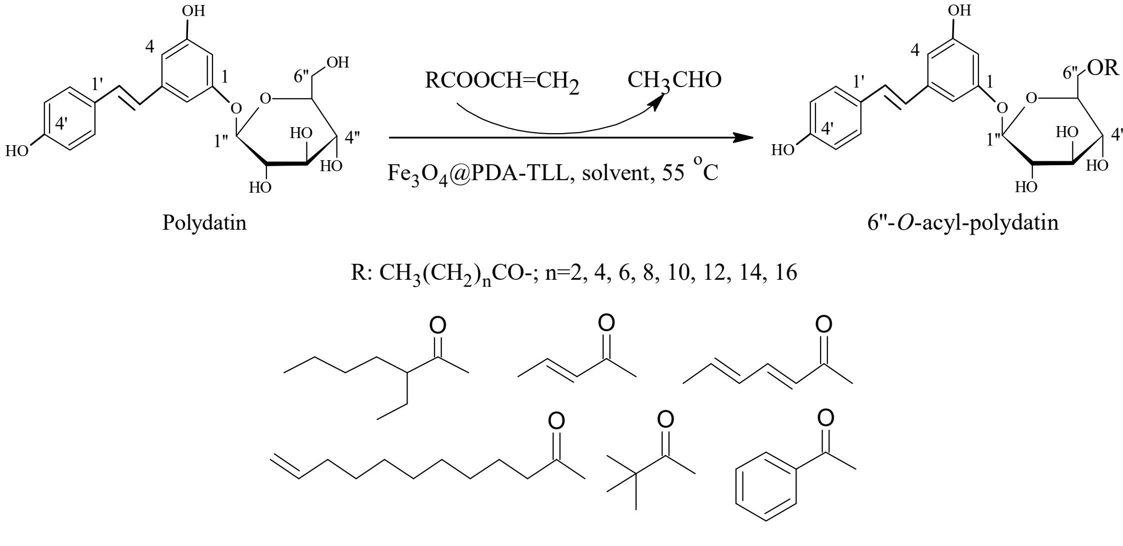

Fig. 1 Enzymatic regioselective acylation of polydatin with various acyl donors.

6''-*O*-Butyryl-polydatin (95% isolated yield, EA/PE=1/1). ^{1}H NMR (DMSO-d_6) δ: 9.59 (br s, 1H, OH phenolic), 9.45 (br s, 1H, OH phenolic), 7.41 (d, 2H *J*=5.6 Hz, H-2'+H-6'), 7.02 (d, 1H, *J*=10.8 Hz, H-vinyl-1), 6.87 (d, 1H, *J*=10.8 Hz, H-vinyl-2), 6.77 (d, 1H *J*=5.6 Hz, H-3'), 6.65 (br s, 1H, H-5'), 6.59 (br s, 1H, H-2), 6.31 (t, 2H, *J*=1.2 Hz, H-6+H-4), 5.36 (br s, 1H, OH-2''), 5.29 (d, 1H, *J*=2.8 Hz, OH-3''), 5.20 (br s, 1H, OH-4''), 4.89 (d, 1H, *J*=5.2 Hz, H-1''), 4.34 (d, 1H, *J*=9.2, H-6''-1), 4.07 (dd, 1H, *J*=5.2, 4.8 Hz, H-6''-2), 3.63 (m, 1H, H-5''), 3.31-3.24 (m, 1H, H-2''), 3.24 (br s, 1H, H-3''), 3.17-3.14 (m, 1H, H-4''), 2.51 (m, 2H, H-2'''), 2.24-2.21 (m, 2H, H-3'''), 0.75 (t, 3H, *J*=4.8 Hz, H-4'''). ^{13}C NMR (DMSO-d_6) δ: 173.16 (C-1'''), 159.03 (C-3), 158.87 (C-5), 157.79 (C-4'), 139.78 (C-1), 128.98 (C-2'+C-6'), 128.41 (C-1'), 128.36 (Cvinyl-1), 125.73 (Cvinyl-2), 115.99 (C-3'+C-5'), 107.48 (C-6), 104.07 (C-2), 103.26 (C-1''), 100.53 (C-4), 76.84 (C-2''), 74.21 (C-5''), 73.61 (C-3''), 70.56 (C-4''), 63.89 (C-6''), 35.76 (C-2'''), 18.32 (C-3'''), 13.75 (C-4''').

6''-*O*-Crotonyl-polydatin (88% isolated yield, EA/PE=1/1). ^{1}H NMR (DMSO-d_6) δ: 9.63 (br s, 1H, OH phenolic), 9.51 (br s, 1H, OH phenolic), 7.44 (d, 2H *J*=6.0 Hz, H-2'+H-6'), 7.07 (d, 1H, *J*=8.0 Hz, H-vinyl-1), 6.93 (d, 1H, *J*=4.0 Hz, H-3'''), 6.92 (br s, 1H, H-2'''), 6.88-6.87 (m, 1H, H-vinyl-2), 6.82 (d, 1H, *J*=5.6 Hz, H-3'), 6.71 (br s, 1H, H-5'), 6.64 (br s, 1H, H-2), 6.37 (t, 1H, *J*=1.6 Hz, H-6), 5.85 (dd, 1H, *J*=1.2, 1.2 Hz, H-4), 5.42 (d, 1H, *J*=3.6 Hz, OH-2''), 5.36 (d, 1H, *J*=3.6 Hz, OH-3''), 5.26 (d, 1H, *J*=2.0 Hz, OH-4''), 4.95 (d, 1H, *J*=5.2 Hz, H-1''), 4.47 (d, 1H, *J*=6.8, H-6''-1), 4.11 (dd, 1H, *J*=4.8, 5.2 Hz, H-6''-2), 3.74-3.71 (m, 1H, H-5''), 3.39-3.36 (m, 1H, H-2''), 3.33-3.29 (m, 1H, H-3''), 3.26-3.22 (m, 1H, H-4''), 1.68 (dd, 3H, *J*=0.8, 1.2 Hz, H-4'''). ^{13}C NMR (DMSO-d_6) δ: 165.94 (C-1'''), 159.04 (C-3), 158.88 (C-5), 157.81 (C-4'), 145.88 (C-3'''), 139.79 (C-1), 128.96 (C-2'+C-6'), 128.42 (C-1'), 128.39 (Cvinyl-1), 125.70 (Cvinyl-2), 122.46 (C-2'''), 116.04 (C-3'+C-5'), 107.63 (C-6), 104.82 (C-2), 103.27 (C-1''), 100.50 (C-4), 76.88 (C-2''),

74.20 (C-5''), 73.60 (C-3''), 70.61 (C-4''), 64.03 (C-6''), 17.94 (C-4''').

6''-*O*-Hexanoyl-polydatin (92% isolated yield, EA/PE=3/2). ^{1}H NMR (DMSO-d_6) δ: 9.59 (br s, 1H, OH phenolic), 9.46 (br s, 1H, OH phenolic), 7.40 (d, 2H *J*=5.6 Hz, H-2'+H-6'), 7.03 (d, 1H, *J*=11.2 Hz, H-vinyl-1), 6.87 (d, 1H, *J*=11.2 Hz, H-vinyl-2), 6.77 (d, 1H *J*=4.8 Hz, H-3'), 6.66 (br s, 1H, H-5'), 6.60 (br s, 1H, H-2), 6.32 (t, 2H, *J*=1.2 Hz, H-6+H-4), 5.38 (br s, 1H, OH-2''), 5.30 (d, 1H, *J*=2.0 Hz, OH-3''), 5.22 (br s, 1H, OH-4''), 4.89 (d, 1H, *J*=5.2 Hz, H-1''), 4.35 (d, 1H, *J*=8.0, H-6''-1), 4.07 (dd, 1H, *J*=4.4, 7.2 Hz, H-6''-2), 3.65-3.63 (m, 1H, H-5''), 3.33-3.31 (m, 1H, H-2''), 3.26 (br s, 1H, H-3''), 3.19-3.17 (m, 1H, H-4''), 2.51 (m, 2H, H-2'''), 2.25-2.21 (m, 2H, H-3'''), 1.44-1.39 (m, 2H, H-4'''), 1.16-1.07 (m, 2H, H-5'''), 0.76 (t, 3H, *J*=4.4 Hz, H-6'''). ^{13}C NMR (DMSO-d_6) δ: 172.60 (C-1'''), 159.05 (C-3), 158.86 (C-5), 157.85 (C-4'), 139.82 (C-1), 129.86 (C-2'+C-6'), 128.95 (C-1'), 128.40 (Cvinyl-1), 125.76 (Cvinyl-2), 116.05 (C-3'+C-5'), 107.62 (C-6), 104.79 (C-2), 103.37 (C-1''), 100.55 (C-4), 76.89 (C-2''), 74.29 (C-5''), 73.62 (C-3''), 70.59 (C-4''), 64.17 (C-6''), 33.86 (C-2'''), 31.63 (C-4'''), 21.18 (C-3'''), 18.77 (C-5'''), 14.52 (C-6''').

6''-*O*-Sorboyl-polydatin (89% isolated yield, EA/PE=3/2). ^{1}H NMR (DMSO-d_6) δ: 9.63 (br s, 1H, OH phenolic), 9.51 (br s, 1H, OH phenolic), 7.43 (d, 2H, *J*=6.0 Hz, H-2'+H-6'), 7.18-7.13 (m, 1H, *J*=18.0 Hz, H-4'''), 7.05 (d, 1H, *J*=10.8 Hz, H-vinyl-1), 6.91 (d, 1H, *J*=10.8 Hz, H-3'''), 6.88 (t, 1H, *J*=2.4 Hz, H-2'''), 6.87 (t, 1H, H-vinyl-2), 6.81 (d, 1H, *J*=5.6 Hz, H-3'), 6.70 (br s, 1H, H-5'), 6.63 (br s, 1H, H-2), 6.36 (t, 1H, *J*=1.6 Hz, H-6), 6.16-6.12 (m, 1H, H-5'''), 5.80 (dd, 1H, *J*=1.2, 1.2 Hz, H-4), 5.42 (br s, 1H, OH-2''), 5.36 (br s, 1H, OH-3''), 5.24 (br s, 1H, OH-4''), 4.94 (d, 1H, *J*=5.2 Hz, H-1''), 4.47 (d, 1H, *J*=7.2, H-6''-1), 4.11 (dd, 1H, *J*=5.2, 5.2 Hz, H-6''-2), 3.74-3.71 (m, 1H, H-5''), 3.39-3.36 (m, 1H, H-2''), 3.32-3.30 (m, 1H, H-3''), 3.25-3.22 (m, 1H, H-4''), 1.71 (d, 3H, *J*=4.4 Hz, H-6'''). ^{13}C NMR (DMSO-d_6) δ: 166.77 (C-1'''), 159.05 (C-3), 158.86 (C-5), 157.85

(C-4'), 145.64 (C-3'''), 140.43(C-4'''), 139.82 (C-1), 129.86 (C-2'+C-6'), 128.95 (C-5'''+C-1'), 128.40 (Cvinyl-1), 125.76 (Cvinyl-2), 118.75 (C-2'''), 116.05 (C-3'+C-5'), 107.62 (C-6), 104.79 (C-2), 103.37 (C-1''), 100.55 (C-4), 76.89 (C-2''), 74.29 (C-5''), 73.62 (C-3''), 70.59 (C-4''), 64.17 (C-6''), 18.77 (C-6''').

6''-*O*-Octanoyl-polydatin (87% isolated yield, EA/PE=3/4). ^{1}H NMR (DMSO-d_6) δ: 9.58 (br s, 1H, OH phenolic), 9.44 (br s, 1H, OH phenolic), 7.40 (d, 2H *J*=5.6 Hz, H-2'+H-6'), 7.03 (d, 1H, *J* =11.2 Hz, H-vinyl-1), 6.87 (d, 1H, *J*=11.2 Hz, H-vinyl-2), 6.77 (d, 1H *J*=5.6 Hz, H-3'), 6.66 (br s, 1H, H5'), 6.59 (br s, 1H, H-2), 6.32 (t, 2H, *J*=1.2 Hz, H-6+H-4), 5.37 (d, 1H, *J*=3.6 Hz, OH-2''), 5.30 (d, 1H, *J*=3.6 Hz, OH-3''), 5.21 (d, 1H, *J*=3.2 Hz, OH-4''), 4.88 (d, 1H, *J*=5.2 Hz, H-1''), 4.34 (d, 1H, *J*=6.8, H-6''-1), 4.07 (dd, 1H, *J*=5.2, 5.2 Hz, H-6''-2), 3.65-3.62 (m, 1H, H-5''), 3.33-3.30 (m, 1H, H-2''), 3.27-3.25 (m, 1H, H-3''), 3.19-3.17 (m, 1H, H-4''), 2.51 (m, 2H, H-2'''), 2.25-2.22 (m, 2H, H-3'''), 1.42-1.40 (m, 2H, H-4'''), 1.20-1.08 (m, 6H, H-5'''+H-6'''+H-7'''), 0.81 (t, 3H, *J*=4.8 Hz, H-8'''). ^{13}C NMR (DMSO-d_6) δ: 173.32 (C-1'''), 159.02 (C-3), 158.87 (C-5), 157.82 (C-4'), 139.78 (C-1), 128.96 (C-2'+C-6'), 128.39 (C-1'), 128.33 (Cvinyl-1), 125.70 (Cvinyl-2), 115.98 (C-3'+C-5'), 107.54 (C-6), 104.92 (C-2), 103.30 (C-1''), 100.52 (C-4), 76.87 (C-2''), 74.19 (C-5''), 73.60 (C-3''), 70.62 (C-4''), 63.92 (C-6''), 33.88 (C-2'''), 31.55 (C-6'''), 28.77 (C-4'''+C-5'''), 24.85 (C-3'''), 22.48 (C-7'''), 14.39 (C-8''').

6''-*O*-Decanoyl-polydatin (85% isolated yield, EA/PE=3/4). ^{1}H NMR (DMSO-d_6) δ: 9.57 (br s, 1H, OH phenolic), 9.44 (br s, 1H, OH phenolic), 7.40 (d, 2H *J*=5.6 Hz, H-2'+H-6'), 7.02 (d, 1H, *J*=10.8 Hz, H-vinyl-1), 6.86 (d, 1H, *J*=10.8 Hz, H-vinyl-2), 6.76 (d, 1H *J*=7.6 Hz, H-3'), 6.65 (br s, 1H, H-5'), 6.58 (br s, 1H, H-2), 6.31 (t, 2H, *J*=1.2 Hz, H-6+H-4), 5.36 (d, 1H, *J*=3.2 Hz, OH-2''), 5.29 (d, 1H, *J*=3.2 Hz, OH-3''), 5.20 (d, 1H, *J*=2.8 Hz, OH-4''), 4.87 (d, 1H, *J*=5.2 Hz, H-1''), 4.33

(d, 1H, *J*=6.8, H-6''-1), 4.06 (dd, 1H, *J*=5.2, 5.2 Hz, H-6''-2), 3.64-3.61 (m, 1H, H-5''), 3.30-3.29 (m, 1H, H-2''), 3.26-3.23 (m, 1H, H-3''), 3.18-3.15 (m, 1H, H-4''), 2.51 (m, 2H, H-2'''), 2.24-2.21 (m, 2H, H-3'''), 1.42-1.39 (m, 2H, H-4'''), 1.24-1.11 (m, 10H, H-5'''+H-6'''+H-7'''+H-8'''+H-9'''), 0.83 (t, 3H, *J*=4.4 Hz, H-10'''). ^{13}C NMR (DMSO-d_6) δ: 173.32 (C-1'''), 159.02 (C-3), 158.87 (C-5), 157.82 (C-4'), 139.76 (C-1), 128.94 (C-2'+C-6'), 128.38 (C-1'), 128.32 (C-vinyl-1), 125.69 (C-vinyl-2), 115.97 (C-3'+C-5'), 107.51 (C-6), 104.94 (C-2), 103.28 (C-1''), 100.53 (C-4), 76.87 (C-2''), 74.19 (C-5''), 73.60 (C-3''), 70.62 (C-4''), 63.92 (C-6''), 33.88 (C-2'''), 31.74 (C-8'''), 29.30-28.83 (C-4'''+C-5'''+C-6'''+C-7'''), 24.85 (C-3'''), 22.56 (C-9'''), 14.43 (C-10''').

6''-*O*-Undecylenoyl-polydatin (90% isolated yield, EA/PE=3/4). ^{1}H NMR (DMSO-d_6) δ: 9.58 (br s, 1H, OH phenolic), 9.45 (br s, 1H, OH phenolic), 7.40 (d, 2H *J*=6.0 Hz, H-2'+H-6'), 7.03 (d, 1H, *J*=10.8 Hz, H-vinyl-1), 6.88 (s, 1H, H-vinyl-2), 6.86 (br s, 1H, H-3'), 6.78 (d, 1H, *J*=6.0 Hz, H-5'), 6.67 (br s, 1H, H-2), 6.37 (t, 1H, *J*=1.6 Hz, H-6), 5.77-5.74 (m, 2H, H-4+H-10'''), 5.39 (d, 1H, *J*=3.6 Hz, OH-2''), 5.31 (d, 1H, *J*=3.6 Hz, OH-3''), 5.23 (d, 1H, *J*=2.0 Hz, OH-4''), 4.99-4.88 (m, 3H, H-1''+H-11'''), 4.35 (d, 1H, *J*=6.8, H-6''-1), 4.09 (dd, 1H, *J*=4.8, 5.2 Hz, H-6''-2), 3.66-3.63 (m, 1H, H-5''), 3.34-3.32 (m, 1H, H-2''), 3.30-3.27 (m, 1H, H-3''), 3.21-3.18 (m, 1H, H-4''), 2.51 (t, 2H, *J*=1.2, H-2'''), 2.25-2.22 (m, 2H, H-9'''), 1.98-1.95 (m, 2H, H-3'''), 1.42-1.10 (m, 10H, H-4'''+H-5'''+H-6'''+H-7'''+H-8'''). ^{13}C NMR (DMSO-d_6) δ: 173.33 (C-1'''), 159.03 (C-3), 158.88 (C-5), 157.83 (C-4'), 139.78 (C-10'''), 139.32 (C-1), 128.96 (C-2'+C-6'), 128.38 (C-1'), 128.31 (C-vinyl-1), 125.69 (C-vinyl-2), 115.99 (C-11'''), 115.00 (C-3'+C-5'), 107.54 (C-6), 104.97 (C-2), 103.31 (C-1''), 100.56 (C-4), 76.88 (C-2''), 74.21 (C-5''), 73.61 (C-3''), 70.65 (C-4''), 63.93 (C-6''), 33.89 (C-9'''), 33.66 (C-2'''), 29.17-28.74 (C-4'''+C-5'''+C-6'''+C-7'''+C-8'''), 24.85 (C-3''').

6''-*O*-Lauroyl-polydatin (91% isolated yield, EA/PE=2/5). ^{1}H NMR (DMSO-d_6) δ: 9.57 (br s,

1H, OH phenolic), 9.45 (br s, 1H, OH phenolic), 7.39 (d, 2H J=5.6 Hz, H-2'+H-6'), 7.02 (d, 1H, J=11.2 Hz, H-vinyl-1), 6.86 (d, 1H, J=11.2 Hz, H-vinyl-2), 6.77 (d, 1H J=6.0 Hz, H-3'), 6.66 (br s, 1H, H-5'), 6.61 (br s, 1H, H-2), 6.34 (t, 2H, J=1.2 Hz, H-6+H-4), 5.40 (d, 1H, J=3.2 Hz, OH-2''), 5.32 (d, 1H, J=3.2 Hz, OH-3''), 5.23 (d, 1H, J=2.0 Hz, OH-4''), 4.89 (d, 1H, J=5.2 Hz, H-1''), 4.35 (d, 1H, J=6.8, H-6''-1), 4.09 (dd, 1H, J=5.2, 5.2 Hz, H-6''-2), 3.66-3.63 (m, 1H, H-5''), 3.34-3.33 (m, 1H, H-2''), 3.30-3.28 (m, 1H, H-3''), 3.20-3.19 (m, 1H, H-4''), 2.51 (m, 2H, H-2'''), 2.24-2.22 (m, 2H, H-3'''), 1.42-1.40 (m, 2H, H-4'''), 1.25-1.10 (m, 14H, H-5'''+H-6'''+H-7'''+H-8'''+H-9'''+H-10'''+H-11'''), 0.83 (t, 3H, J=4.4 Hz, H-12'''). ^{13}C NMR (DMSO-d_6) δ: 173.31 (C-1'''), 159.03 (C-3), 158.88 (C-5), 157.83 (C-4'), 139.77 (C-1), 128.95 (C-2'+C-6'), 128.37 (C-1'), 128.28 (C-vinyl-1), 125.67 (C-vinyl-2), 115.98 (C-3'+C-5'), 107.52 (C-6), 105.01 (C-2), 103.31 (C-1''), 100.58 (C-4), 76.88 (C-2''), 74.21 (C-5''), 73.60 (C-3''), 70.65 (C-4''), 63.92 (C-6''), 33.90 (C-2'''), 31.80 (C-10'''), 29.51-28.87 (C-4'''+C-5'''+C-6'''+C-7'''+C-8'''+C-9'''), 24.86 (C-3'''), 22.59 (C-11'''), 14.40 (C-12''').

6''-*O*-Myristoyl-polydatin (87% isolated yield, EA/PE=2/5). ^{1}H NMR (DMSO-d_6) δ: 9.57 (br s, 1H, OH phenolic), 9.44 (br s, 1H, OH phenolic), 7.39 (d, 2H J=5.6 Hz, H-2'+H-6'), 7.02 (d, 1H, J=11.2 Hz, H-vinyl-1), 6.86 (d, 1H, J=11.2 Hz, H-vinyl-2), 6.77 (d, 1H J=6.0 Hz, H-3'), 6.66 (br s, 1H, H-5'), 6.60 (br s, 1H, H-2), 6.33 (t, 2H, J=1.2 Hz, H-6+H-4), 5.39 (d, 1H, J=3.6 Hz, OH-2''), 5.31 (d, 1H, J=3.6 Hz, OH-3''), 5.23 (d, 1H, J=2.8 Hz, OH-4''), 4.88 (d, 1H, J=5.2 Hz, H-1''), 4.35 (d, 1H, J=6.8, H-6''-1), 4.09 (dd, 1H, J=5.2, 5.2 Hz, H-6''-2), 3.66-3.62 (m, 1H, H-5''), 3.34-3.32 (m, 1H, H-2''), 3.30-3.27 (m, 1H, H-3''), 3.21-3.18 (m, 1H, H-4''), 2.51 (m, 2H, H-2'''), 2.25-2.22 (m, 2H, H-3'''), 1.42-1.40 (m, 2H, H-4'''), 1.26-1.11 (m, 18H, H-5'''+H-6'''+H-7'''+H-8'''+H-9'''+H-10'''+H-11'''+H-12'''+H-13'''), 0.84 (t, 3H, J=4.4 Hz, H-14''').

^{13}C NMR (DMSO-d_6) δ: 173.29 (C-1'''), 159.04 (C-3), 158.88 (C-5), 157.84 (C-4'), 139.76 (C-1), 128.94 (C-2'+C-6'), 128.36 (C-1'), 128.27 (C-vinyl-1), 125.67 (C-vinyl-2), 115.97 (C-3'+C-5'), 107.52 (C-6), 105.01 (C-2), 103.30 (C-1''), 100.60 (C-4), 76.89 (C-2''), 74.21 (C-5''), 73.60 (C-3''), 70.64 (C-4''), 63.93 (C-6''), 33.89 (C-2'''), 31.81 (C-12'''), 29.54-28.88 (C-4'''+C-5'''+C-6'''+C-7'''+C-8'''+C-9'''+C-10'''+C-11'''), 24.86 (C-3'''), 22.60 (C-13'''), 14.39 (C-14''').

6''-*O*-Palmitoyl-polydatin (85% isolated yield, EA/PE=2/5). ^{1}H NMR (DMSO-d_6) δ: 9.56 (br s, 1H, OH phenolic), 9.43 (br s, 1H, OH phenolic), 7.39 (d, 2H *J*=5.6 Hz, H-2'+H-6'), 7.02 (d, 1H, *J*=11.2 Hz, H-vinyl-1), 6.86 (d, 1H, *J*=11.2 Hz, H-vinyl-2), 6.76 (d, 1H *J*=5.6 Hz, H-3'), 6.65 (br s, 1H, H-5'), 6.59 (br s, 1H, H-2), 6.32 (t, 2H, *J*=1.2 Hz, H-6+H-4), 5.37 (d, 1H, *J*=3.2 Hz, OH-2''), 5.29 (d, 1H, *J*=3.2 Hz, OH-3''), 5.21 (d, 1H, *J*=2.8 Hz, OH-4''), 4.87 (d, 1H, *J*=5.2 Hz, H-1''), 4.34 (d, 1H, *J*=7.6, H-6''-1), 4.08 (dd, 1H, *J*=5.2, 5.2 Hz, H-6''-2), 3.65-3.61 (m, 1H, H-5''), 3.33-3.30 (m, 1H, H-2''), 3.27-3.24 (m, 1H, H-3''), 3.19-3.15 (m, 1H, H-4''), 2.51 (m, 2H, H-2'''), 2.24-2.21 (m, 2H, H-3'''), 1.43-1.40 (m, 2H, H-4'''), 1.27-1.11 (m, 22H, H-5'''+H-6'''+H-7'''+H-8'''+H-9'''+H-10'''+H-11'''+H-12'''+H-13'''+H-14'''+H-15'''), 0.85 (t, 3H, *J*=4.8 Hz, H-16'''). ^{13}C NMR (DMSO-d_6) δ: 173.29 (C-1'''), 159.03 (C-3), 158.88 (C-5), 157.83 (C-4'), 139.75 (C-1), 128.93 (C-2'+C-6'), 128.37 (C-1'), 128.28 (C-vinyl-1), 125.67 (C-vinyl-2), 115.96 (C-3'+C-5'), 107.50 (C-6), 104.99 (C-2), 103.28 (C-1''), 100.57 (C-4), 76.88 (C-2''), 74.20 (C-5''), 73.60 (C-3''), 70.63 (C-4''), 63.93 (C-6''), 33.88 (C-2'''), 31.78 (C-14'''), 29.55-28.86 (C-4'''+C-5'''+C-6'''+C-7'''+C-8'''+C-9'''+C-10'''+C-11'''+C-12'''+C-13'''), 24.86 (C-3'''), 22.58 (C-15'''), 14.40 (C-16''').

6''-*O*-Stearoyl-polydatin (85% isolated yield, EA/PE=2/5). ^{1}H NMR (DMSO-d_6) δ: 9.55 (br s,

1H, OH phenolic), 9.42 (br s, 1H, OH phenolic), 7.38 (d, 2H *J*=6.0 Hz, H-2'+H-6'), 7.02 (d, 1H, *J* =10.8 Hz, H-vinyl-1), 6.85 (d, 1H, *J*=10.8 Hz, H-vinyl-2), 6.76 (d, 1H *J*=6.0 Hz, H-3'), 6.66 (d, 1H, *J*=6.0 Hz, H-5'), 6.59 (br s, 1H, H-2), 6.33 (t, 2H, *J*=1.2 Hz, H-6+H-4), 5.38 (d, 1H, *J*=2.0 Hz, OH-2''), 5.30 (br s, 1H, OH-3''), 5.22 (br s, 1H, OH-4''), 4.87 (d, 1H, *J*=2.0 Hz, H-1''), 4.34 (d, 1H, *J*=6.8, H-6''-1), 4.09 (dd, 1H, *J*=5.2, 5.2 Hz, H-6''-2), 3.65-3.62 (m, 1H, H-5''), 3.35-3.32 (m, 1H, H-2''), 3.28-3.26 (m, 1H, H-3''), 3.20-3.17 (m, 1H, H-4''), 2.51 (t, 2H, *J*=1.2, H-2'''), 2.24-2.15 (m, 2H, H-3'''), 1.49-1.40 (m, 2H, H-4'''), 1.26-1.11 (m, 26H, H-5'''+H-6'''+H-7'''+H-8'''+H-9'''+H-10'''+H-11'''+H-12'''+H-13'''+H-14'''+H-15'''+H-16'''+H-17'''), 0.84 (t, 3H, *J*=4.4 Hz, H-18'''). ^{13}C NMR (DMSO-d_6) δ: 173.24 (C-1'''), 159.05 (C-3), 158.89 (C-5), 157.85 (C-4'), 139.74 (C-1), 128.92 (C-2'+C-6'), 128.35 (C-1'), 128.24 (C-vinyl-1), 125.66 (C-vinyl-2), 115.95 (C-3'+C-5'), 107.51 (C-6), 105.01 (C-2), 103.29 (C-1''), 100.63 (C-4), 76.89 (C-2''), 74.20 (C-5''), 73.60 (C-3''), 70.63 (C-4''), 63.93 (C-6''), 33.88 (C-2'''), 31.83 (C-16'''), 29.61-28.92 (C-4'''+C-5'''+C-6'''+C-7'''+C-8'''+C-9'''+C-10'''+C-11'''+C-12'''+C-13'''+C-14'''+C-15'''), 24.87 (C-3'''), 22.60 (C-17'''), 14.34 (C-18''').

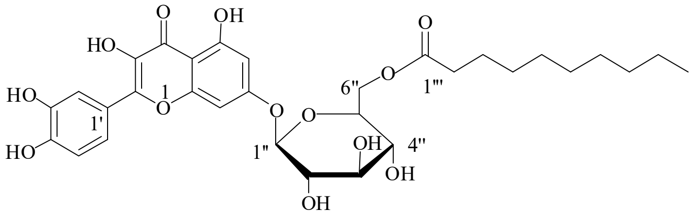

Fig. 2 Enzymatic regioselective acylation of quercimetrin with vinyl decanoate.

6''-*O*-Decanoyl-quercimetrin (90% isolated yield, EA/PE=1/5). ^{1}H NMR (DMSO-d_6) δ: 12.63 (1H, s, 5-OH), 7.64 (1H, dd, *J*=8.5, 2.2 Hz, H-6'), 7.49 (1H, d, *J*=2.2 Hz, H-2'), 6.81 (1H, d, *J*=8.5 Hz, H-5'), 6.39 (1H, d, *J*=2.0 Hz, H-8), 6.18 (1H, d, *J*=2.0 Hz, H-6), 4.90 (1H, d, *J*=3.6 Hz, H-1''), 5.21 (1H, br s, -OH), 4.95 (1H, br s, -OH), 4.69 (1H, br s, -OH), 4.11 (1H, dd, *J*=11.4, 8.4 Hz,

H-5"), 3.91 (1H, dd, *J*=11.4, 3.8 Hz, H-6"a), 3.61 (1H, m, H-4"), 3.40-3.50 (3H, m, H-3",6”b, H-2"), 1.93-2.00 (2H, m, H-2'''), 1.01-1.24 (14H, m, H-3'''-9'''), 0.86 (3H, t, *J*=7.1 Hz, H-10'''). ^{13}C NMR (DMSO-d_6) δ: 156.6 (C-2), 133.7 (C-3), 177.9 (C-4), 161.7 (C-5), 99.1 (C-6), 164.6 (C-7), 93.9 (C-8), 156.6 (C-9), 104.2 (C-10), 121.5 (C-1'), 116.2 (C-2'), 145.3 (C-3'), 148.9 (C-4'), 115.6 (C-5'), 122.4 (C-6'), 98.3 (C-1"), 75.4 (C-2"), 71.5 (C-3"), 68.9 (C-4"), 71.9 (C-5"), 63.6 (C-6 "). 172.9 (C-1'''), 33.7 (C-2'''), 31.8 (C-8'''), 28.8-29.3 (C-4'''-7'''), 24.7 (C-3'''), 22.6 (C-9'''), 14.4 (C-10''').

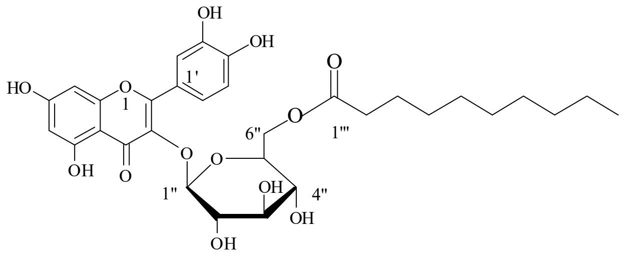

Fig. 3 Enzymatic regioselective acylation of isoquercetin with vinyl decanoate.

6"-*O*-Decanoyl-isoquercetin (87% isolated yield, EA/PE=5/15). ^{1}H NMR (DMSO-d_6) δ: 12.62 (1H, s, 5-OH), 7.52 (1H, m, H-6,2'), 6.82 (1H, d, *J*=8.5 Hz, H-5'), 6.39 (1H, s, H-8), 6.19 (1H, s, H-6), 5.44 (1H, d, *J*=7.8 Hz, H-1"), 5.37 (1H, br s, -OH), 4.18 (2H, br s, -OH), 4.14 (1H, dd, *J*=11.4Hz, H-5"), 3.94 (1H, dd, *J*=11.6, 7.1 Hz, H-6"a), 3.12-3.31 (3H, m, H-3",6"b, H-2",4"), 1.94-2.00 (2H, m, H-2'''), 1.01-1.24 (14H, m, H-3'''-9'''), 0.86 (3H, t, *J*=6.9 Hz, H-10'''). ^{13}C NMR (DMSO-d_6) δ: 156.8 (C-2), 133.4 (C-3), 177.8 (C-4), 161.7 (C-5), 99.1 (C-6), 164.6 (C-7), 93.9 (C-8), 156.8 (C-9), 104.3 (C-10), 121.5 (C-1'), 116.5 (C-2'), 145.3 (C-3'), 148.9 (C-4'), 115.5 (C-5'), 122.0 (C-6'), 101.1 (C-1"), 74.6 (C-2"), 76.7 (C-3"), 70.5 (C-4"), 74.4 (C-5"), 63.4 (C-6 ").,172.9 (C-1'''), 33.7 (C-2'''), 31.8 (C-8'''), 28.8-29.3 (C-4'''-7'''), 24.7 (C-3'''), 22.6 (C-9'''), 14.1 (C-10''').

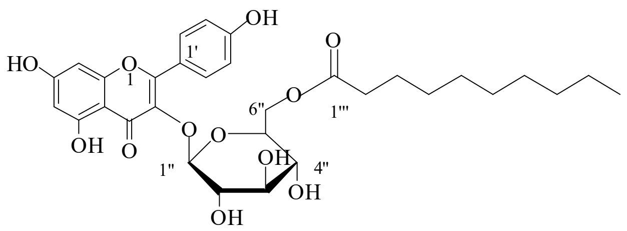

Fig. 4 Enzymatic regioselective acylation of astragaline with vinyl decanoate.

6''-*O*-Decanoyl-astragaline (88% isolated yield, EA/PE=15/5). ^{1}H NMR (DMSO-d_6) δ: 12.58 (1H, s, 5-OH), 8.00 (2H, d, *J*=8.9 Hz, H-6',2'), 6.88 (2H, d, *J*=8.9 Hz, H-3',5'), 6.42 (1H, d, *J*=2.0 Hz, H-8), 6.21 (1H, d, *J*=2.0 Hz, H-6), 5.43 (1H, d, *J*=7.6 Hz, H-1''), 5.21 (1H, br s, -OH), 4.15 (1H, dd, *J*=11.4Hz, H-5''), 3.99 (1H, dd, *J*=11.8, 6.9 Hz, H-6''a), 3.13-3.35 (3H, m, H-3'',6''b, H-2'',4''), 1.94-2.00 (2H, m, H-2'''), 1.01-1.24 (14H, m, H-3'''-9'''), 0.84 (3H, t, *J*=6.9 Hz, H-10'''). ^{13}C NMR (DMSO-d_6) δ: 156.8 (C-2), 133.4 (C-3), 177.8 (C-4), 161.7 (C-5), 99.2 (C-6), 164.7 (C-7), 94.0 (C-8), 156.9 (C-9), 104.3 (C-10), 121.2 (C-1'), 131.2 (C-2',6'), 115.5 (C-3',5'), 160.5 (C-4'), 101.4 (C-1''), 74.5 (C-2''), 76.7 (C-3''), 70.5 (C-4''), 74.5 (C-5''), 63.3 (C-6'').,172.8 (C-1'''), 33.7 (C-2'''), 31.8 (C-8'''), 28.8-29.3 (C-4'''-7'''), 24.7 (C-3'''), 22.6 (C-9'''), 14.1 (C-10''').

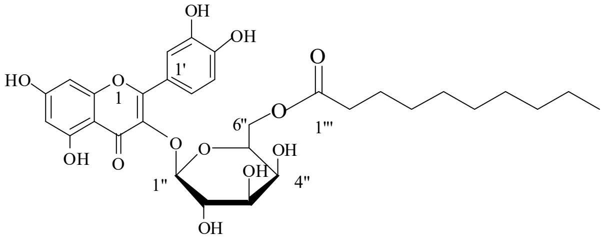

Fig. 5 Enzymatic regioselective acylation of hyperoside with vinyl decanoate.

6''-*O*-Decanoyl-hyperoside (90% isolated yield, EA/PE=10/5). ^{1}H NMR (DMSO-d_6) δ: 12.63 (1H, s, 5-OH), 7.64 (1H, dd, *J*=8.5, 2.2 Hz, H-6’), 7.49 (1H, d, *J*=2.2 Hz, H-2'), 6.81 (1H, d, *J*=8.5 Hz, H-5'), 6.39 (1H, d, *J*=2.0 Hz, H-8), 6.18 (1H, d, *J*=2.0 Hz, H-6), 5.38 (1H, d, *J*=7.8 Hz, H-1''), 5.21 (1H, br s, -OH), 4.95 (1H, br s, -OH), 4.69 (1H, br s, -OH), 4.11 (1H, dd, *J*=11.4, 8.4 Hz, H-5''), 3.91 (1H, dd, *J*=11.4, 3.8 Hz, H-6''a), 3.61 (1H, m, H-4''), 3.40-3.50 (3H, m, H-3'',6''b,

H-2''), 1.93-2.00 (2H, m, H-2'''), 1.01-1.24 (14H, m, H-3'''-9'''), 0.86 (3H, t, J=7.1 Hz, H-10'''). ^{13}C NMR (DMSO-d_6) δ: 156.6 (C-2), 133.7 (C-3), 177.9 (C-4), 161.7 (C-5), 99.1 (C-6), 164.6 (C-7), 93.9 (C-8), 156.6 (C-9), 104.2 (C-10), 121.5 (C-1'), 116.2 (C-2'), 145.3 (C-3'), 148.9 (C-4'), 115.6 (C-5'), 122.4 (C-6'), 101.8 (C-1''), 71.4 (C-2''), 73.4 (C-3''), 68.8 (C-4''), 75.0 (C-5''), 63.6 (C-6 ''). 172.9 (C-1'''), 33.7 (C-2'''), 31.8 (C-8'''), 28.8-29.3 (C-4'''-7'''), 24.7 (C-3'''), 22.6 (C-9'''), 14.4 (C-10''').

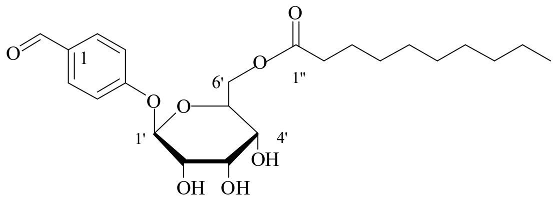

Fig. 6 Enzymatic regioselective acylation of helicid with vinyl decanoate.

6''-*O*-Decanoyl-helicid (91% isolated yield, EA/PE=2/3). ^{1}H NMR (DMSO-d_6) δ: 9.89 (s, 1, OH7), 7.87 (d, 2, J=8.3 Hz, H3+ H5), 7.17 (d, 2, J=8.4 Hz, H2+ H6), 5.26 (t, 2, J=7.2 Hz, OH2''+ OH3''), 5.15 (apparent d, 1, J=3.8 Hz, OH4''), 4.97 (d, 1, J=7.4 Hz, H1''), 4.32 (d, 1, J=11.6 Hz, H6''), 4.10 (dd, 1, J=11.7, 7.1 Hz, H6''), 3.98-4.04 (m, 2, H4''+ H5''), 3.51-3.55 (m, 2, H2''+ H3''), 2.27 (t, 2, J=7.4 Hz, H2'), 1.47 (p, 2, J=7.1 Hz, H3'), 1.18-1.21 (m, 12, H4'+ H5'+ H6'+ H7'+ H8'+ H9'), 0.83 (t, 3, J=6.8 Hz, H12'), ^{13}C NMR: (DMSO-d_6) δ: 191.16 (C7), 172.67 (C1'), 162.10 (C1), 131.47 (C3, 5), 130.49 (C4), 116.26 (C2, 6), 97.84 (C1''), 71.54 (C3''), 71.26 (C5''), 69.98 (C2''), 67.32 (C4''), 63.58 (C6''), 33.49 (C2'), 31.22 (C8'), 28.80 (C5'), 28.67 (C6'), 28.60 (C7'), 28.43 (C4'), 24.40 (C3'), 22.03 (C9'), 13.83 (C10').

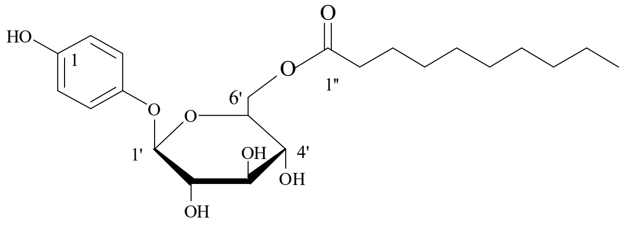

Fig. 7 Enzymatic regioselective acylation of arbutin with vinyl decanoate.

6'-*O*-Decanoyl-arbutin (95% isolated yield, EA/PE=1/4). ^{1}H NMR (DMSO-d_6) δ: 9.00 (s, 1H, OH-4), 6.84 (apparent, d, 2H, H-2+H-6), 6.66 (apparent, d, 2H, H-3+H-6), 5.30 (d, 1H, *J*=4.9 Hz, OH-4'), 5.23 (d, 1H, *J*=5.4 Hz, OH-3'), 4.67 (d, 1H, *J*=7.5 Hz, H-1'), 4.33 (d, 1H, *J*=10.8 Hz, H-6'), 4.15 (d, 1H, *J*=4.6 Hz, OH-4'), 4.08 (dd, 1H, *J*=7.2, 11.8 Hz, H-6'), 3.50 (t, 1H, *J*=8.2 Hz, H-5'), 3.29-3.09 (m, 3H, H-2',H-3', H-4'), 2.28 (t, 2H, *J*=7.3 Hz, H-2''), 1.51 (t, 2H, *J*=6.8 Hz, H-3''), 1.23 (s, 12H, H-4'', H-5'', H-6'', H-7'', H-8'', H-9''), 0.85 (t, 3H, *J*=6.7 Hz, H-10''). ^{13}C NMR (DMSO-d_6) δ: 172.6 (C-1''), 152.2 (C-4), 150.0 (C-1), 117.5 (C-2-6), 115.3(C-3), 101.4 (C-1'), 76.2 (C-5'), 73.5 (C-3'), 73.1 (C-2'), 69.9 (C-4'), 63.3 (C-6'), 33.4 (C-2''), 31.2 (C-8''), 28.7 (C-6''), 28.6 (C-7''), 28.5 (C-5''), 28.4 (C-4''), 24.32 (C-3''), 21.9 (C-9''), 13.9 (C-10'').

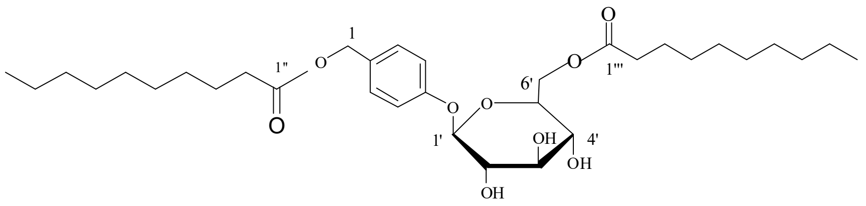

Fig. 8 Enzymatic regioselective acylation of gastrodin with vinyl decanoate.

1,6'-*O*-Decanoyl-gastrodin (93% isolated yield, EA/PE=3/1). ^{1}H NMR (DMSO-d_6) *δ*: 7.28 (2H, d, *J*=8.7 Hz, H-3,7), 6.99 (2H, d, *J*=8.7 Hz, H-4,6), 5.39 (1H, d, *J*=5.1 Hz, H-1'), 5.29 (1H, d, *J*=5.5 Hz, -OH), 5.21 (1H, d, *J*=3.5 Hz, -OH), 5.01 (2H, s, H-1), 4.89 (1H, d, *J*=7.6 Hz, -OH), 4.33 (1H, dd, *J*=11.8, 2.0 Hz, H-5'), 4.05 (1H, dd, *J*=11.8, 7.0 Hz, H-6'), 3.61 (1H, m, H-4'), 3.24-3.32 (2H, m, H-3',6'), 3.14-3.18 (1H, m, H-2'), 2.26-2.32 (4H, m, H-2'',2'''), 1.49-1.54 (4H, m, H-3'',3'''), 1.23-1.24 (24H, m, H-4''-9'', H-4'''-9'''), 0.84-0.87 (6H, m, H-10'',10'''). ^{13}C NMR (DMSO-d_6) *δ*:173.1 (C-1'''), 173.1 (C-1''), 157.4 (C-5), 130.0 (C-3,7), 130.0 (C-2), 116.5 (C-4,6), 100.5 (C-1'), 76.7 (C-5'), 74.1 (C-3'), 73.5 (C-2'), 70.4 (C-4'), 65.4 (C-1), 63.8 (C-6'), 34.0 (C-2''), 33.9 (C-2'''), 31.5 (C-8''), 31.5 (C-8'''), 28.8-28.9 (C-4'''-7''', C-4''-7''), 24.8 (C-3''), 24.8 (C-3'''), 22.5 (C-9''), 22.4 (C-9'''), 14.3 (C-10''), 14.3 (C-10''').

Reference

1. Yoshimoto, K., Itatani, Y., Tsuda, Y.. 13C-Nuclear magnetic resonance spectra of *O*-acylglucose. Additivity of shift parameters and its application to structure elucidations. Chem. Pharm. Bull. 1980, 28, 2065-2074.

2. Li, N., Zong, M.H., Ma, D.. Regioselective acylation of nucleosides and their analogs catalyzed by *Pseudomonas cepacia* lipase: enzyme substrate recognition. Tetrahedron. 2009, 65: 1063-1068.
